# A Breath of Fresh Air on the Mesenchyme: Impact of Impaired Mesenchymal Development on the Pathogenesis of Bronchopulmonary Dysplasia

**DOI:** 10.3389/fmed.2015.00027

**Published:** 2015-04-28

**Authors:** Cho-Ming Chao, Elie El Agha, Caterina Tiozzo, Parviz Minoo, Saverio Bellusci

**Affiliations:** ^1^Department of General Pediatrics and Neonatology, University Children’s Hospital Giessen, Giessen, Germany; ^2^Department of Internal Medicine II, Universities of Giessen and Marburg Lung Center, Giessen, Germany; ^3^Member of the German Center for Lung Research (DZL), Giessen, Germany; ^4^Division of Neonatology, Department of Pediatrics, Columbia University, New York, NY, USA; ^5^Division of Newborn Medicine, Department of Pediatrics, Children’s Hospital Los Angeles, University of Southern California, Los Angeles, CA, USA; ^6^Saban Research Institute, Childrens Hospital Los Angeles, University of Southern California, Los Angeles, CA, USA; ^7^Kazan Federal University, Kazan, Russia

**Keywords:** lung development, alveologenesis, bronchopulmonary dysplasia, epithelial–mesenchymal interaction, endothelial–mesenchymal interaction, secondary septae formation

## Abstract

The early mouse embryonic lung, with its robust and apparently reproducible branching pattern, has always fascinated developmental biologists. They have extensively used this embryonic organ to decipher the role of mammalian orthologs of *Drosophila* genes in controlling the process of branching morphogenesis. During the early pseudoglandular stage, the embryonic lung is formed mostly of tubes that keep on branching. As the branching takes place, progenitor cells located in niches are also amplified and progressively differentiate along the proximo-distal and dorso-ventral axes of the lung. Such elaborate processes require coordinated interactions between signaling molecules arising from and acting on four functional domains: the epithelium, the endothelium, the mesenchyme, and the mesothelium. These interactions, quite well characterized in a relatively simple lung tubular structure remain elusive in the successive developmental and postnatal phases of lung development. In particular, a better understanding of the process underlying the formation of secondary septa, key structural units characteristic of the alveologenesis phase, is still missing. This structure is critical for the formation of a mature lung as it allows the subdivision of saccules in the early neonatal lung into alveoli, thereby considerably expanding the respiratory surface. Interruption of alveologenesis in preterm neonates underlies the pathogenesis of chronic neonatal lung disease known as bronchopulmonary dysplasia. *De novo* formation of secondary septae appears also to be the limiting factor for lung regeneration in human patients with emphysema. In this review, we will therefore focus on what is known in terms of interactions between the different lung compartments and discuss the current understanding of mesenchymal cell lineage formation in the lung, focusing on secondary septae formation.

## Bronchopulmonary Dysplasia is Characterized by Impaired Alveologenesis

Bronchopulmonary dysplasia (BPD) is a chronic lung disease of prematurely born infants and remains a leading cause of morbidity and mortality. Currently, there is no curative therapy available. Based on the severity-based definition of BPD (inclusion of infants with mild BPD) 68% of premature infants born with a gestational age (GA) ≤28 weeks develop BPD (1–3). The risk of developing BPD correlates inversely with the GA and birth weight (BW) (4). Since premature infants (24–28 weeks of gestation) are born with a lung, which is in the canalicular or saccular stages of development, the lung structure (characterized by thickened airspace walls and surfactant deficiency) is therefore not adequate to provide sufficient ventilation and gas exchange. Thus, mechanical ventilation and high-oxygen concentration are often necessary at birth. Barotrauma induced by mechanical ventilation as well as oxygen toxicity and inflammation are major contributing factors responsible for the pulmonary damages in the morphological and functional immature lung. In addition, some studies have suggested a strong genetic component in BPD (5). For example, using genome-wide association study, it has been shown that polymorphisms (SNPs) in *MMP16* and *SPOCK2* might be associated with BPD (6). Due to remarkable advances in the management and therapy (e.g., gentle ventilation, restricted oxygen supplementation, antenatal steroids, and exogenous surfactant use) survival rate for premature infants has increased over the last decades. These advances in treatment have changed the histological characteristics of what is now called the old BPD since it was first described by Northway in 1967. The “old” BPD was mostly an airway disease characterized by interstitial fibrosis and squamous metaplasia of airways. The prominent histological findings in the lungs of “new” BPD are simplification of alveolar formation (fewer and larger alveoli) and dysmorphic pulmonary microvasculature (7, 8). Pulmonary hypertension is also a common complication in infants with BPD, resulting in high mortality (9). According to these findings, the “new” BPD is considered as a consequence of the premature lung interrupted in its development by postnatal lung injury leading to the growth arrest of the lung in the canalicular/saccular phase of normal lung development. BPD, as a chronic lung disease, leads to long-term morbidity (e.g., pulmonary infection, neurodevelopmental impairment) affecting quality of life during childhood and in some severely affected patients even into adulthood. Treatment for BPD represents a considerable health care burden (10–12).

The mechanisms responsible for alveolar simplification in BPD remain understudied and poorly understood. However, autopsy samples from premature infants from pre- and post-surfactant era, who died from BPD consistently showed abnormalities in the mesenchyme (interstitial fibrosis and dysmorphic microvasculature). In the new BPD, there is clear evidence for decreased number of secondary septae, a derivative of the lung mesoderm. Furthermore, animal models mimicking the premature lung and the risk factors for BPD provide more evidence that indeed the mesenchyme plays a pivotal role in late lung development/alveologenesis and therefore in BPD. This review will summarize the current understanding of the impaired mesenchymal compartment of the BPD lungs, with a focus on mesenchymal–endothelial and mesenchymal–epithelial crosstalk known to contribute to disease pathogenesis.

## Normal Lung Development in Human and Mouse

In human and mouse, the lung arises from two germ layers: the gut endoderm gives rise to the lung epithelium and the splanchnic mesoderm is the origin of the lung mesenchyme. The human lung consists of three lobes on the right and two lobes on the left side; in mice four lobes form on the right (cranial, medial, caudal, and accessory lobe) and one on the left. Compared to the 12 airway generations observed in mice, human lungs comprise 23 airway generations.

In humans, lung development arises from the laryngo-tracheal groove and starts at week 4 of gestation as an outgrowth from the ventral wall of the caudal primitive foregut. During the further growth of the lung, the prospective trachea separates from the foregut by the formation of the so-called tracheo-esophageal septum. At the most distal part of the tracheal tube, two buds that will form the right and left primary bronchial buds appear. These primary buds are further ramified to form three secondary bronchial buds on the right and two secondary bronchial buds on the left side. These buds are the origin of the five lobes in the mature lung (13).

**Figure 1:**
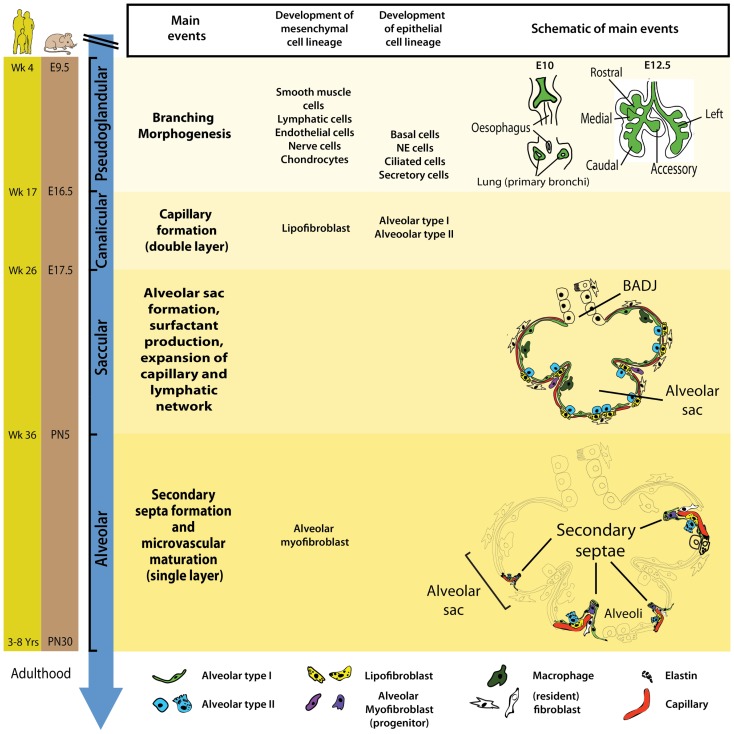
**Timeline and stages of lung development in mice and humans**. Lung development starts with the specification of the lung domain in the foregut endoderm followed by the formation of primary lung buds. These buds will later give rise to the respiratory tree via the process of branching morphogenesis. The latter is a characteristic of the pseudoglandular stage of lung development. Most epithelial and mesenchymal cell types start to form during the pseudoglandular stage. The canalicular stage is characterized by blood capillary formation and the appearance of AECI/II. During the saccular stage, primitive alveoli (sac-like structures) start to form and this is accompanied by surfactant production and the expansion of capillary and lymphatic networks. The alveolar stage of lung developments starts *in utero* in humans whereas in mice, it starts postnatally. Wk, week; E, embryonic; PN, postnatal; NE, neuroendocrine.

In mice, at embryonic day 8 (E8), signaling molecules and growth factors (e.g., Fgf1, Fgf2) emanate from the cardiac mesoderm and specify the prospective lung field in the primitive foregut endoderm, which is positive for the transcription factor *Nkx2.1* (or *Ttf1*). These pre-lung epithelial progenitor cells represent the earliest and most likely the most pluripotent epithelial cells for the lung. At E9.5, the ventral foregut endoderm evaginates and elongates caudally dividing into two buds that form the prospective trachea and the first generation of bronchi (main bronchi). The process of lung development (human and mouse) has been divided into four distinct histological phases: pseudoglandular, canalicular, saccular, and alveolar (Figure 1).

During the pseudoglandular stage (human: week 4–17; mouse: E9.5–E16.5), the process of branching morphogenesis generates the basic tree-like structure of the lung including the conducting airways and the numerous terminal bronchioles surrounded by thick mesenchyme. Concurrently, epithelial cell progenitors undergo differentiation to give rise to basal, neuroendocrine, ciliated, and secretory cells. The mesodermal lung compartment serves as progenitors for the smooth muscle, lymphatic, endothelial, nerve, and chondrocytic cells.

In the subsequent canalicular stage (human: week 17–26; mouse: E16.5–E17.5), the lung undergoes further subdivision of the respiratory bronchioles accompanied by thinning of the surrounding mesenchyme and the massive formation of capillaries. For the first time during development, a primitive respiratory epithelium competent of gas exchange is formed by differentiation of distal lung epithelial progenitors. Recently, it has been shown that type I and type II alveolar epithelial cells (AEC I and II) emerge from a common alveolar bipotential progenitor (14). In mice, interstitial fibroblasts containing cytoplasmic lipid droplets (so called lipofibroblast, LIF) emerge in the mesenchyme. Additionally, this is the earliest time point of pregnancy (23–24 weeks of gestation) where a preterm infant can be born with a chance to survive. Those who died from BPD showed pathologic characteristics of the lung (interstitial fibrosis and dysmorphic microvasculature) similar to the morphology of the immature lung at this developmental stage thus reinforcing the concept that BPD results from interruption of normal lung development by deleterious environmental events. The introduction of antenatal steroids treatment and exogenous surfactant supplementation drastically increased survival of premature infants born at this stage (15).

The saccular stage of lung development occurs approximately between 26 and 36 weeks of gestation (mouse: E17.5–PN5). This stage is characterized by the formation of alveolar sacs, surfactant production, and thinning of the mesenchyme to facilitate gas exchange. Kresch demonstrated that the thinning of the mesenchyme results from apoptosis of mesenchymal cells (16). Furthermore, the capillary and lymphatic networks also expand in the saccular stage of lung development.

**Figure 2:**
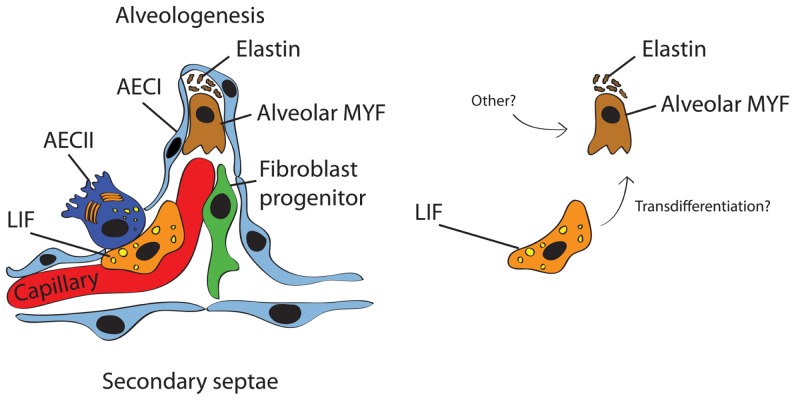
**Schematic representation of the secondary septum during alveologenesis**. Most of the alveolar surface is occupied by AECI (gas exchange) whereas a minor surface is occupied by AECII (surfactant production). The alveolar wall consists of the blood capillary, LIF, resident fibroblast progenitor, alveolar MYF, and ECM (mostly elastin). It has been proposed that alveolar MYF can originate from LIF (right panel) but this concept needs further validation.

The last stage of lung development is termed alveolar stage (human: ~36 weeks to 8 years; mouse: PN5–PN30). During this stage, the alveolar surface area increases massively at the expense of the mesenchyme through subdividing the alveolar sacs (also called primitive alveoli) into mature alveoli by a process termed alveolarization (or alveologenesis) (Figure 2). This process starts with the deposition of elastin in primary septae (wall of alveolar sacs) and subsequently secondary septae emerge at the place of elastin and elongate toward the alveolar sac airspace to subdivide it into the smallest respiratory units of the lung – the mature alveoli. Importantly, concomitant with this process, primary septae, still containing a double layer of capillaries, become thinner and a single capillary network emerges allowing more efficient gas exchange (microvascular maturation). The bulk of alveolarization takes place during the first 6 months after birth in humans (mouse: PN5–PN15) (17). The alveolar myofibroblast (MYF), localized in the mesenchyme at the tip of the emerging secondary septae, is the cell responsible for secondary septae formation. A more detailed description of this mesenchymal cell lineage will be provided in the following sections.

In summary, the lung is a complex ramified organ that develops through continuous and elaborate interaction among the epithelium, mesenchyme, mesothelium, and endothelium. During this process, an intricate signaling network controls the amplification, proliferation, migration, and differentiation of diverse progenitor cells to populate these different compartments. Importantly, most of the epithelial and mesenchymal cell types in the lung are formed during the late pseudoglandular stage (E13.5–E16.6). This means that any deleterious factors present prenatally (such as inflammations due to chorioamnionitis) or postnatally (such as barotrauma injury and subsequent inflammation due to oxygen or mechanical ventilation), interfering with normal lung development at that time, could lead to impaired pulmonary function postnatally. Since preterm infants who die from BPD commonly display abnormal mesenchyme, a better understanding of aberrant signaling pathways in the lung mesenchyme of BPD lungs is important for improving the existing, and may facilitate the development of new preventive and curative therapies. In the next section, the current knowledge, mostly obtained from animal models of BPD, about abnormalities occurring in the lung mesenchyme will be reviewed.

## The Embryonic Lung Mesenchyme

During the pseudoglandular stage of lung development (~E13.5), the distal lung bud is composed of three morphologically distinguishable layers: the mesothelium (outer layer), the mesenchyme (middle layer), and the epithelium (inner layer). The mesenchyme can be further divided into two domains, the submesothelial mesenchyme (SMM) and the subepithelial mesenchyme (SEM). Whereas mesenchymal cells constituting the SEM display high density and circumferential orientation, those of the SMM display low density and organization. Lineage-tracing experiments have identified markers for some mesenchymal progenitors such as Wnt2/Gli1/Isl1 (originating from the heart and invading the lung), Ret, Pdgfrα, Vegfr2, Prox1, and Fgf10 (18–20). Progenitors in these two compartments give rise to various cell types such as airway smooth muscle cells (ASMCs), vascular smooth muscle cells (VSMCs), resident mesenchymal stem cells (MSCs), LIFs, endothelial cells, chondrocytes, nerve cells, alveolar MYFs, lymphatic cells, and others. Mesenchymal progenitor cells are believed to play important roles not only in development but also in homeostasis and regeneration after injury.

### Epithelial–mesenchymal crosstalk in normal lung development and BPD

During development, the lung is formed through an elaborated epithelial–mesenchymal crosstalk that drives lung specification, budding, and branching. Signaling molecules like fibroblast growth factors (Fgf), Wnt (wingless and int), Sonic hedgehog (Shh), and bone morphogenetic proteins (Bmp) are key ligands initiating the pulmonary cell fate and specifying the early lung domain at the ventral foregut endoderm (21). So far, the most convincing evidence for epithelial–mesenchymal interactions during lung development came from recombination studies where distal lung mesenchyme, grafted on the tracheal epithelium led to ectopic budding accompanied by expression of surfactant protein C as a distal epithelial marker (22–24).

The mammalian Fgf family consists of 22 members subdivided in 7 subfamilies, based on phylogenetic as well as gene loci analyses (25). Fgfs acts in a paracrine, endocrine, or intracrine fashion and have diverse biological activities during embryonic organogenesis. These growth factors act via seven main receptors (Fgfrs 1b, 1c, 2b, 2c, 3b, 3c, and 4), exhibiting different ligand-binding specificity. The Fgf receptors are encoded by four *Fgfr* genes (*Fgfr1–Fgfr4*), which undergo alternative splicing to produce the different isoforms. Each receptor comprises an extracellular ligand-binding domain with three immunoglobulin-like loops (D I, D II, D III), a transmembrane domain and an intracellular tyrosine kinase domain. Human diseases involving gain or loss of function mutations have been described. For example, loss of function of *FGF3* causes deafness, heterozygous loss of function of *FGF10* results in lacrimo-auriculo-dento-digital syndrome (LADD syndrome), *FGF10* haploinsufficiency is also associated with chronic obstructive pulmonary disease and *FGF23* gain of function leads to autosomal dominant hypophosphataemic rickets (26–29). During early (E12.5) embryonic mouse lung development, Fgf9 and Fgf10 have been shown to play an important role in branching morphogenesis and the associated differentiation of the epithelium and mesenchyme. Fgf9 is expressed in the mesothelium and the epithelium and acts through Fgfr2c- and Fgfr1c-expressing cells in the mesenchyme to maintain Fgf10 expression as well as mesenchymal progenitors proliferative and undifferentiated (30). It also can signal directly to the epithelium to promote epithelial branching by induction of *Dkk1* expression and inhibition of Wnt signaling (31). Fgf10 is a diffusible key molecule orchestrating branching morphogenesis during early lung development in mice (32, 33) but the exact mechanism of action remains unknown. During the early pseudoglandular stage, Fgf10 is secreted by cells located adjacent to the mesothelium in the distal mesenchyme and signals in a paracrine manner mainly through fibroblast growth factor receptor 2-IIIb (Fgfr2b) expressed on epithelial cells. Fgf10 has a high affinity for heparan sulfate and is therefore unlikely to diffuse over a long distance. Instead, Fgf10 promotes outgrowth of the distal epithelium via a chemotactic mechanism. Several studies using transgenic mouse lines that display abnormal Fgf10/Fgfr2b signaling confirmed the importance of this pathway (Table 1). *Fgf10* and *Fgfr2b* knockout pups display similar phenotypes. The mutant pups die shortly after birth due to lung agenesis and multiple organ agenesis/defects (salivary gland, limb, inner ear, teeth, skin, pancreas, kidney, thyroid, pituitary gland, mammary gland) (34–38).

**Table 1:** **Overview of proteins that are known to be involved in alveologenesis**.

Protein name	Origin	Localization/targets	Function in alveologenesis	Alterations in BPD	Alterations in animal model of BPD	Effect of genetic modulation in the animal model
Elastin	Alveolar myofibroblast	Tip of growing secondary septae	Secondary septae formation (tips)	Increased and disorganized in saccular walls (66, 67)	Decreased in hyperoxia (133)	KO: inhibited alveolarization (87)
Pdgfa	Epithelial cells, macrophages	Pdgfrα-expressing cells (ASMC, alv. MYF, LIF)	Chemotactic attractant for fibroblasts (134)	Not known	Delayed in hyperoxia (135)	KO: inhibited alveolarization (93, 94)
Fgf10	Mesenchymal cells located in SMM	Distal epithelial cells expressing Fgfr2b	Under investigation	Decreased (75)	Decreased in LPS-model (76)	KO: lung agenesis
						Partial deficiency: delayed/disturbed lung branching (41)
Tgf-β/Tgf-β1	Epithelial cells	Epithelial and mesenchymal cells	Modulation of cell survival, differentiation and ECM (Elastin) deposition (136, 137)	Increased in tracheal aspirate (138)	Increased in hyperoxia (139, 140)	Overexpression: inhibition of branching morphogenesis and alveolarization (141)
						Inhibition: attenuated hyperoxia-induced hypoalveolarization (140)
Vegf	Epithelial (during embryonic development also in mesenchymal cells)	Endothelial cells (Vegfr1/2)	Stimulation of endothelial cells for angio-/vasculogenesis (essential for alveolarization)	Decreased (8, 127)	Decreased in hyperoxia (125); (126)	Inhibition: hypoalveolarization (142, 143)

In order to identify epithelial-specific gene expressions mediated by recombinant human FGF10 during bud morphogenesis, Lu and colleagues (39) used mesenchyme-free epithelium in culture. By using microarray analysis, they identified a panel of transcriptional *Fgf10* targets, which are associated with cell rearrangement, migration, inflammatory processes, lipid metabolism, cell cycle, and tumor invasion. Interestingly, the authors did not observe a remarkable induction of genes responsible for proliferation. Moreover, Fgf10 is proposed to control the angle of the mitotic spindle in distal epithelial cells during development. Thus, Tang et al. argued that Fgf10 signals via a Ras-regulated Erk1/2 signaling pathway to shape the lung tube (40). Fgf10 is also critical for the amplification of distal epithelial cell progenitors and for the formation of multiple mesenchymal lineages during lung development. Hypomorphic *Fgf10^lacZ/^*^−^ pups expressing ~20% *Fgf10* compared to wild type (WT) died within 24–48 h after birth due to lung defects, which included decreased branching, thickened primary septae, and vascular abnormalities with intrapulmonary hemorrhages. At the cellular level, *Fgf10* deficiency led to decrease in Nkx2.1 and Sftpb-expressing cells, suggesting that adequate *Fgf10* expression level is critical for the amplification of epithelial progenitors. Apart from the epithelium, constitutive decrease in *Fgf10* expression also affects mesenchymal cell lineages as *Pecam* and α*Sma*-positive cells are also diminished (41). Interestingly, recent experiments conducted in our lab to investigate the impact of *Fgf10* levels on lung function demonstrate that even a 50% decrease in *Fgf10* expression (*Fgf10* heterozygous pups) leads to changes in the expression of genes relevant for lung development such as *Epcam, Sftpc*, *Fgfr2b*, *Tgf*-β, and *Collagen*. Additionally, *Fgf10* heterozygous neonatal mice survive and do not display any obvious phenotypic differences compared to WT mice. However, when exposed to hyperoxia between PN0 and PN8 to trigger lung injury and mimic some of the clinical manifestations of BPD (impaired alveologenesis and inflammation), *Fgf10*-deficient pups display drastically increased mortality compared to WT controls. Further analysis indicates that under physiological conditions, *Fgf10*-deficient mice already show structural abnormalities during embryonic lung development supporting that *Fgf10*-deficient pups carry congenital defects. These findings suggest that *Fgf10*-deficient lung epithelium is more susceptible to oxygen toxicity and does not undergo normal repair after injury (Chao and Bellusci, in preparation). Additionally, recent studies suggest that Fgf10 may control basal cell density in the tracheal epithelium (42–45). This is not surprising as it has already been previously shown, by our group and others, that Fgf10 is part of the stem cell niche in the lung (20, 46, 47).

Wnt (Wnt2, Wnt2b) ligands are expressed in the mesenchyme and are important for lung domain specification of the foregut endoderm from E9.0 to E10.5. Wnt signaling is also essential for the proximo-distal patterning of the epithelium during embryonic lung development. Genetic deletion of *Wnt2/2b* or β*-catenin* leads to lung agenesis due to loss of *Nkx2.1* (48, 49). *Wnt2* null mice display lung hypoplasia and abnormal development of ASMCs (50). Furthermore, Mucenski and colleagues demonstrated, by using *Spc-rtTA;tet(O)Cre* double transgenic mice, that loss of function of β*-catenin* in the distal lung epithelium leads to the inhibition of distal airway formation (51). The authors showed an opposite phenotype by inducing gain of function of β*-catenin* signaling (52). The absence of *Wnt7b* results in a phenotype similar to *Wnt2* null mice (53) but a combination of *Wnt7b* and *Wnt2* loss of function leads to a more severe phenotype with decreased branching and abnormal distal endoderm patterning (54). The constitutive deletion of *Wnt5a* – a non-canonical Wnt ligand expressed in the mesenchyme and the epithelium – leads to increased proliferation of the mesenchyme and the distal epithelium as well as disrupted lung maturation (55). β*-catenin* inactivation in the mesenchyme (*Dermo1-Cre* line) leads to abnormal mesenchyme development with disrupted amplification of ASMC progenitors and defects in angioblast differentiation (56). Kumar and colleagues demonstrated by using a clonal cell labeling approach that ASMC progenitors are located exclusively at the tip mesenchyme and that mesenchymal Wnt signaling is able to prime the stalk mesenchyme to form an ASMC progenitor pool at the tip (57).

*Bmp4* is dynamically expressed in the endoderm and in the mesenchyme during early embryonic lung development (E11.5). It is also expressed at the distal epithelial buds and has been shown to be an inhibitor of Fgf10-induced chemotaxis in the epithelium. Bmp4 controls intraepithelial crosstalk to form ASMCs. It has been shown that *Fgf10* is able to upregulate *Bmp4* mRNA expression. *In vitro* experiments demonstrated that exogenous recombinant human BMP4 inhibits Fgf10-induced bud outgrowth, providing evidence that Bmp4 is acting downstream of Fgf10 to inhibit its signaling cascade (58–61).

Other Fgf10 inhibitors are Sonic hedgehog (Shh) and Sprouty homolog 2 (Spry2), both expressed in the epithelium of the outgrowing buds. Shh is a secreted growth factor that acts through its mesenchymal receptor Patched (Ptc) to induce mesenchymal cell proliferation and differentiation. In E11.5 lung explants, exogenous recombinant SHH is able to induce expression of mesenchymal markers (*Noggin*, *Acta2*, *Myosin*) (19, 62). Spry2 is an intracellular inhibitor of receptor tyrosine kinase signaling (63, 64); (32). Using *in vitro* approaches, it has been shown that *Spry2* reduction leads to increased epithelial branching and vice versa.

Apart from its important role in development, the mesenchyme is crucial in disease pathogenesis. Indeed, it has been reproducibly shown that the lung mesenchyme in preterm infants dying from BPD includes interstitial fibrosis and thickening with increased total collagen content (65–67). Similar findings were obtained in diverse animal models (rat, mice, baboon) recapitulating the conditions of preterm infants after birth (mechanical ventilation, oxygen supplementation, exogenous surfactant) leading to a human BPD-like phenotype (68–72). These pathological changes in the lung mesenchyme in BPD strengthen the concept that alveolarization depends on an intact and normally developed mesenchyme. Several studies using animal models of BPD to identify molecules located in the altered lung mesenchyme contributed to our understanding of disease pathogenesis. Some of them will be reviewed in the following section.

One of the major causes of BPD is believed to be inflammation. Inflammation is caused prenatally by chorioamnionitis and postnatally by mechanical stretch (ventilation), oxygen toxicity, as well as infection. Emerging evidence gained from *in vitro* and *in vivo* studies support this hypothesis (73–77). For example, it has been shown that lipopolysaccharides (LPS from *Escherichia coli*) inhibit branching morphogenesis *in vitro* (73). Blackwell et al. published similar results using activated resident macrophages to inhibit epithelial branching. The proposed mechanism is that LPS activates nuclear factor kappa beta (NF-kappa B), which is then accompanied by increased expression of *interleukin-1beta* (*IL-1*β) and *tumor necrosis factor-a* (*TNF-a*) in resident macrophages (74). This branching inhibitory effect caused by macrophage-mediated inflammation has been confirmed by a macrophage-depletion study in the lung. Benjamin et al. explained this inhibitory effect by linking Fgf10 signaling with inflammatory signals. Using *in vitro* experiments, they demonstrated that NF-kappa B, IL-1β, and TNF-*a* are capable of reducing *Fgf10* expression in LPS-treated primary mesenchymal cells. The mechanism involved activation of toll-like receptors 2 and 4 (TLR2/4). The authors showed that FGF10-positive cells were decreased in lung samples of premature infants who died from BPD (75).

Tgf-β1 has been demonstrated to induce epithelial–mesenchymal transition (EMT) of AEC to MYF-like cells leading to extracellular matrix (ECM) deposition and thereby contributing to fibrosis and destruction of alveolar structure (78–80) (see also Table 1). Endogenous nitric oxide is proposed to attenuate EMT in AECs in an *in vitro* approach using primary culture of AEC II (81).

As previously mentioned, the alveologenesis phase leads to a dramatic increase in alveolar surface, which is essential for gas exchange. The current consensus is that this process is interrupted by exogenous deleterious factors leading to simplification of alveoli in BPD. Many studies confirmed that the alveolar MYF, located in the mesenchyme, is the unique cell type responsible for secondary septae formation. During alveologenesis, the alveolar myofibroblast is characterized by expression of alpha-smooth-muscle-actin (αSMA or Acta2) compared to other mesenchymal fibroblast population. By deposition of elastin and collagen, the alveolar myofibroblast initiates the process of secondary septation (82, 83). Both elastin and alveolar myofibroblast have been shown to be critical for secondary septae formation (83, 84). Expression of tropoelastin starts in the pseudoglandular stage of lung development and reaches the highest level during the alveolar stage (85, 86). The strongest evidence so far showing the importance of elastin for secondary septae formation came from the *Elastin*-knock-out mice that reveal a complete failure of alveologenesis leading to an emphysematous-like phenotype (87, 88) (Table 1). Interestingly, both hyperoxia and mechanical ventilation lead to increased expression of Elastin (89–91). Fgfr3 and Fgfr4 have been shown to direct alveologenesis in the murine lung by controlling elastogenesis (92). By using mice homozygous for *Pdgfa-*null allele, Boström and colleagues demonstrated failed alveolar formation due to loss of alveolar myofibroblasts and consequent loss of Elastin fibers (93, 94). Likewise, blocking antibody against Pdgfrα in newborn mice (PN1–PN7) led to aberrant Elastin fiber deposition and impaired alveolar septation, resulting in long-term failure in alveologenesis that lasted into adulthood. Pdgfa is expressed in the epithelium and targets its receptor (Pdgfrα) on mesenchymal cells such as alveolar myofibroblast and LIF (Table 1). Given the many mesenchymal targets of Pdgfa, it is not clear whether the impact of Pdgfa or Pdgfrα deletion on myofibroblast formation is via a direct effect of Pdgfa on alveolar myofibroblasts (and or alveolar myofibroblast progenitors) or indirectly via Pdgfa action on other targets (ASMCs and LIF). Gain and loss of function for Pdgfa/Pdgfrα signaling using cell autonomous-based approaches in specific lineages should be carried out in the future to sort out these issues.

Interestingly, increased levels of *Fgf* signaling in the mesenchyme also leads to arrested development of terminal airways accompanied by reduced Elastin deposition (95). The authors achieved this condition by taking advantage of *Fgfr2c*^+^*^/Δ^* mice that develop an autocrine Fgf10–Fgfr2b signaling loop in the mesenchyme due to a splicing switch, resulting in the ectopic expression of *Fgfr2b* instead of *Fgfr2c*. The proposed mechanism of action is that mesenchymal *Fgf* signaling suppresses the differentiation of alveolar myofibroblast progenitors. Furthermore, the blockade of Fgfr2b ligands in the lung from E14.5 to E18.5 by overexpression of a soluble dominant negative receptor of *Fgfr2b* (*Sftpc-rtTA/*+*;tetOsolFgfr2b/*+) blocking all Fgfr2b ligands also leads to arrest in secondary septae formation and alveolar simplification (96) suggesting that Fgfr2b ligands, during this time period, are also important for the formation of alveolar myofibroblasts. Subsequent treatment with retinoic acid (RA, biologically active derivative of vitamin A) induced re-alveolarization and was accompanied by increased *Pdgfra*-positive cells and decreased α*Sma/Acta2*-positive cells. Concurrent induction of the dominant negative *Fgfr2b* in these experimental conditions is able to prevent the RA-mediated alveolar regeneration. These data suggest that re-alveolarization is dependent on Fgfr2b ligands. Furthermore, the authors proposed a conceptual model that alveolar myofibroblasts (α*Sma/Acta2*-positive) arise from *Pdgfra*-positive LIF. Specific lineage-tracing studies targeting subsets of lung fibroblasts (e.g., *Adrp* for LIF) are needed to validate this model. Chen and colleagues also demonstrated that Fgfr2b ligands are necessary for alveolar myofibroblast formation during compensatory lung growth after pneumonectomy (97). However, the blockade of Fgfr2b ligands by soluble Fgfr2b (decoy receptor) postnatally during alveolarization does not impair alveologenesis in mice. Recently, it has been shown that reduced *Pdgfra* expression is a primary feature of human BPD. The authors showed decreased mRNA and protein expression of *PDGFR-*α and *PDGFR-*β in MSCs isolated from tracheal aspirates of premature neonates with BPD. Similarly, lungs of infants dying from BPD display less PDGFRα-positive cells in the alveolar septae. These findings were confirmed using a BPD mouse model exposed to hyperoxia (75% oxygen) for 14 days (98).

**Figure 3:**
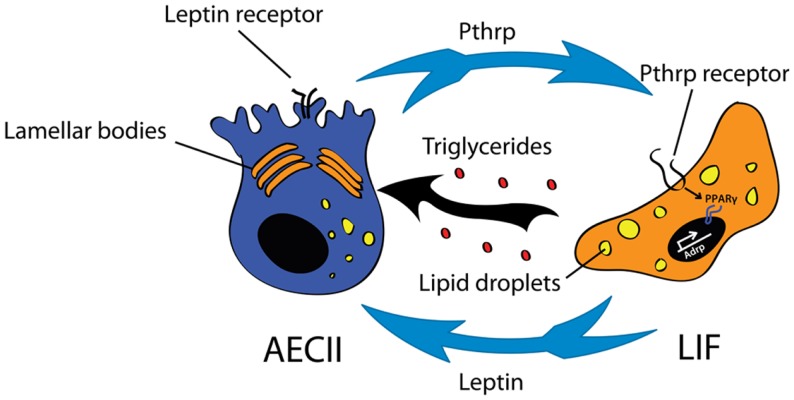
**Interaction between type II alveolar epithelial cells (AEC II) and lipofibroblasts (LIF) for surfactant production**. The Pthrp (parathyroid hormone-related protein)/Pparg (peroxisome proliferator-activated receptor gamma) axis is important for LIF formation and maintenance. LIF secrete triglycerides and leptin that are essential for surfactant production.

The LIF remains a poorly characterized lipid-containing interstitial cell located in the mesenchyme in close proximity to AEC II. LIFs, which accumulate lipid vacuoles (83, 99), are abundant in the early postnatal lung and regress significantly in number after alveolar septation. The presence of LIF in rodent lungs has been demonstrated extensively. However, whether LIF reside in adult human lung remains controversial (100, 101). Because of their close localization to AEC II, LIF have been proposed to interact with AEC II. Indeed, it has been shown convincingly that LIF are involved in the trafficking of lipids to the AEC II for surfactant production (102, 103). Apart from triglycerides, LIF also secrete leptin and retinoic acid, both important for surfactant production and alveolar septation (104, 105). On the other hand, AECII secrete parathyroid hormone-related protein (Pthrp) to signal through Pthrp receptor expressed on LIF to induce expression of adipose differentiation-related protein (*Adrp*) via peroxisome proliferator-activated receptor gamma (Pparg) pathway (Figure 3). The current consensus is that this signaling pathway is essential for the maintenance of the LIF phenotype as well as for regulation of surfactant production (104, 106–108). By performing co-culture experiments it has been proposed recently that LIF constitute a niche for AECII cells postnatally. Co-culture of LIF with AECII cells allows the formation of alveolospheres (109).

The contribution of LIF to lung regeneration and structural maintenance in later phases of life is currently unknown. Several lung injury models including cigarette-smoke exposure induce the transdifferentiation of LIF to αSma/Acta2^+^ MYF *in vitro*. These αSma/Acta2^+^ MYF are highly proliferative and express high levels of collagen (110). For this reason, it has been proposed that LIF are progenitors for alveolar MYF (Figure 2). However, an alternative and more plausible possibility is that LIF give rise to the activated MYF. Activated MYF, unlike the alveolar MYF, is involved in pathological situations and is responsible for fibrosis. Supporting this possibility, we have recently shown that during alveologenesis, *Fgf10*-positive cells give rise to LIF rather than alveolar MYF and during adult life, a subpopulation of *Fgf10*-expressing cells represents a pool of resident MSCs (Cd45^−^ Cd31^−^ Sca-1^+^) (20). In addition, the LIF-to-“activated MYF” transdifferentiation would translate indeed into loss of pulmonary integrity by smoke *in vivo*. Such transdifferentiation can be prevented and reversed *in vitro* using Pparg agonists such as rosiglitazone (111). However, it remains unclear whether such transdifferentiation occurs *in vivo*. Of note, exposure of premature neonates to hyperoxia induces arrest of alveolar septation and thickened primary septae due to MYF hyperplasia and excessive ECM production. Therefore, LIFs are unlikely progenitors for alveolar MYF. In the future, these results will have to be confirmed by lineage-tracing experiments in the context of injury using more specific knock-in lines to target the LIF and determine their fate.

### Endothelial–mesenchymal crosstalk in normal lung development and BPD

In parallel to branching morphogenesis during early embryonic lung development the lung vasculature begins to form in the mesenchyme at around E10.0 (112). This process involves angiogenesis and vasculogenesis. Angiogenesis occurs when pre-existing endothelial cells sprout to form capillaries. In comparison, vasculogenesis is characterized by migration and differentiation of endothelial progenitor cells (or hemangioblasts) in the distal mesenchyme to form new blood vessels. DeMello and colleagues investigated the early fetal development of lung vasculature by employing light and transmission electron microscopy as well as vascular casts and scanning electron microscopy. They demonstrated three features of the lung vasculature occurring between E9.0 and E20.0 in mice: (1) angiogenesis occurs in the proximal (central) lung vasculature, (2) peripheral lung vessels are established by vasculogenesis, and (3) at E13.0/E14.0 the central and peripheral parts of lung vasculature begin to connect to each other via a lytic process (113, 114). Finally, the main event of microvascular maturation takes place during the alveolar stage of lung development where the transition from a double capillary network to a single capillary system within alveolar walls occurs.

Although BPD has long been regarded as an epithelial disease due to its emphysematous aspect, much emphasis has now been placed also on the role of the lung vasculature in this disease. The temporal–spatial proximity of lung vasculature development and branching morphogenesis suggests a close interaction between these two important structures via endothelial–epithelial tissue crosstalk. The better understanding of this crosstalk in development and disease condition might be highly relevant for future therapies. Vascular endothelial growth factor receptor 2 (Vegfr2 or Flk-1) is an early marker for endothelial progenitors located in the SEM (115, 116). However, it is not yet clear whether these progenitors arise from the mesothelium or the mesenchyme (117, 118). Progenitors for VSMCs are believed to arise from Fgf10^+^ cells (20), Wnt2^+^, Gli1^+^ and Isl1^+^ cells (coming from the second heart field) (18), Pdgfrb^+^ cells (119), and mesothelial cells (117, 118).

During murine embryonic development, Vegfr2-positive cells receive the Vegfa signal from epithelial and mesenchymal cells until E14.5, after which *Vegfa* expression becomes restricted to the epithelium (120) (Table 1). Furthermore, it has been shown that Shh and Fgf9, secreted by the epithelium, are able to induce expression of *Vegfa* in the mesenchyme (121). Reciprocally, mesenchymally secreted Fgf10 leads to the upregulation of Vegf in the distal epithelium (122). In our previous work, we showed that treatment of embryonic lung explants with recombinant Vegfa not only upregulates *Vegfr2* in the mesenchyme but also induces branching of the epithelium (123). However, it is unclear whether the effect of Vegfa on epithelial branching is direct or indirect. This endothelial–epithelial tissue crosstalk has been extensively examined by using *in vitro* recombination studies (co-culture of epithelium and mesenchyme respectively and mesenchyme alone) as well as in *in vivo* lung agenesis model (β*-catenin* knockout) (112). Using an *in vivo* inducible decoy receptor of Vegfr1 (solubleVegfr1), Lazarus and colleagues demonstrated that Spry2 is upregulated in the epithelium upon inhibition of Vegfr1-mediated signaling, suggesting an inhibition of Fgf signaling (as mentioned before Spry2 is an inhibitor of Fgf10), which is essential for branching morphogenesis (124). Another link in the endothelial–epithelial crosstalk came from the *Pecam1*-deficient mice that display a failure in endothelial cell formation accompanied by simplified alveolarization (125).

During a pathological process, Vegfa has been found down-regulated in preterm infants with BPD (8, 126, 127). Furthermore, Thebaud and colleagues demonstrated that *Vegf* and *Vegfr2* are decreased in the hyperoxia model of BPD in newborn rats and that adenoviral administration of VEGF improved alveolar architecture and promoted capillary formation (128, 129). Although the trophic and angiogenic potential of VEGF on the lung vasculature is known, the aforementioned study, and the studies from other groups, suggest that vascular growth serves as a driving force for alveolar growth and maturation, leading to improvement of lung structure, and promoting secondary septae formation. A recent report revealed the association of a *VEGF* polymorphism with BPD in Japanese preterm newborns (130).

Newborn mice that are hypomorphic for *Fgf10* also display reduced expression of *Vegfa* and *Pecam*. These mice suffer from an oversimplified lung with an abnormally developed lung vasculature (41). Interestingly, *Fgf10* expression is reduced in lungs from BPD patients (75). Whether the effect of mesenchyme-derived growth factors (such as Fgf10) on the lung endothelium is direct needs to be demonstrated. Another animal injury model demonstrating the importance of endothelial–epithelial interactions is the pneumonectomy model in mice. An inducible endothelium-specific deletion of *Vegfr2* and *Fgfr1* leads to reduction of Mmp14 secretion. Mmp14 is critical for expansion of epithelial progenitor cells during compensatory lung growth by unmasking Egfr ecto-domains. This was confirmed convincingly by rescue-experiments where EGF/MMP14 administration resulted in restored alveologenesis (131).

## Conclusion

Although advances in pharmacotherapy and medical technology (e.g., gentle ventilation) have improved the management of premature infants, BPD remains the most common incurable chronic lung disease of infancy with considerable mortality and long-term morbidity. An unintended consequence of these advances has been the survival rate of premature infants born before the 24th week of gestation who represent the highest risk group for pathogenesis of BPD. This means that the number of infants born with an immature lung (in the canalicular stage of lung development) is increased, leading to an increase in the incidence of BPD. Thus, it is urgent to find a treatment for BPD. The treatment of BPD has been confounded by its multifactorial causes. Therefore, only a comprehensive and individualized therapy adjusted to the profile of risk factors of each prematurely born infant will likely be able to provide a meaningful and effective strategy. The early identification of infants predisposed to BPD is essential. Several studies have been conducted to detect biomarkers in premature infants indicating their level of risk for BPD (132). Yet, the results remain inconclusive due to the low numbers of infants considered for the study. Importantly, more effort should be made in establishing preventive therapy. For example, more research should be conducted to understand the pathogenesis of preterm labor, a common cause of preterm delivery, which is one of the main risk factor associated with BPD. In addition, the current knowledge about the mechanisms of alveolarization must be expanded. Due to lack of human lung samples, the establishment of animal models precisely resembling the disease condition of BPD in humans will be helpful. Single cell transcriptomic studies should be carried out on alveolar myofibroblasts in physiological and pathological conditions to unravel the aberrant gene expression patterns and/or gene mutations responsible for impaired secondary septae formation. Furthermore, the use of the pneumonectomy mouse model and cell specific lineage-tracing approaches to understand the process of *de novo* alveolarization should contribute significantly to our understanding of lung regeneration. Last, but not least, the knowledge about progenitor/stem cells located in niches of the postnatal lung will be a valuable source of information that would be useful in triggering lung regeneration subsequent to injury.

## Conflict of Interest Statement

The authors declare that the research was conducted in the absence of any commercial or financial relationships that could be construed as a potential conflict of interest.
